# A Liver Model of Infantile-Onset Pompe Disease Using Patient-Specific Induced Pluripotent Stem Cells

**DOI:** 10.3389/fcell.2019.00316

**Published:** 2019-11-29

**Authors:** Takeshi Yoshida, Tatsuya Jonouchi, Kenji Osafune, Junko Takita, Hidetoshi Sakurai

**Affiliations:** ^1^Department of Pediatrics, Kyoto University Graduate School of Medicine, Kyoto, Japan; ^2^Center for iPS Cell Research and Application (CiRA), Kyoto University, Kyoto, Japan

**Keywords:** infantile-onset Pompe disease, iPS cell, enzyme replacement therapy, liver, disease modeling

## Abstract

Infantile-onset Pompe disease (IOPD) is a life-threatening multi-organ disease caused by an inborn defect of lysosomal acid α-glucosidase (GAA), which can degrade glycogen into glucose. Lack of GAA causes abnormal accumulation of glycogen in the lysosomes, particularly in the skeletal muscle, liver, and heart. Enzyme replacement therapy (ERT) with recombinant human GAA (rhGAA) is the only available treatment; however, its effect varies by organ. Thus, to fully understand the pathomechanism of IOPD, organ-specific disease models are necessary. We previously generated induced pluripotent stem cells (iPSCs) from three unrelated patients with IOPD and establish a skeletal muscle model of IOPD. Here, we used the same iPSC lines as the previous study and differentiated them into hepatocytes. As a result, hepatocytes differentiated from iPSC of IOPD patients showed abnormal accumulation of lysosomal glycogen, the hallmark of Pompe disease. Using this model, we also demonstrated that glycogen accumulation was dose-dependently restored by rhGAA treatment. In conclusion, we have successfully established an *in vitro* liver model of IOPD using patient-specific iPSCs. This model can be a platform to elucidate the underlying disease mechanism or to be applied to drug-screening. Moreover, our study also suggest that an iPSC-based approach is suitable for modeling of diseases that affect multiple organs like Pompe disease.

## Introduction

Pompe disease (OMIM 232300, glycogen storage disease type II or acid maltase deficiency) is an autosomal recessive disease, caused by an inborn defect of lysosomal acid α-glucosidase (GAA; Hers, 1963). GAA breaks down glycogen into glucose in the lysosomes, and thus the lack of GAA causes abnormal accumulation of glycogen within the lysosomes, particularly in the skeletal muscle, heart, and liver (Kishnani and Howell, 2004). Patients with Pompe disease show a great variety in the severity of their symptoms depending on the residual amount of GAA activity. Patients with complete absence of GAA activities (classified as infantile-onset Pompe disease, IOPD) shows generalized muscle weakness, heart failure, and hepatomegaly in early infancy, and most patients cannot survive over 2 years (Kishnani et al., 2006). The only treatment currently available is enzyme replacement therapy (ERT) with recombinant human GAA (rhGAA), which remarkably reduces the risk of death and invasive ventilation in patients with IOPD (Kishnani et al., 2007). However, ERT is very effective on cardiac and hepatic symptoms; in contrast, its effect on skeletal muscle symptoms is quite limited (Kishnani et al., 2009; Nicolino et al., 2009). This suggests the possibility that each organ has its own pathomechanism. Thus, organ-specific disease models are necessary to fully understand the pathomechanism of IOPD and develop a better therapeutic approach.

Human induced pluripotent stem cells (iPSCs) are very powerful tools to model diseases, especially those affecting multiple organs like IOPD, because of their differentiation potential to various types of tissues (Takahashi et al., 2007). iPSC-based models of various genetic diseases have been established (Rashid et al., 2010; Yoshitoshi-Uebayashi et al., 2017; Sasaki-Honda et al., 2018). In the case of Pompe disease, cardiomyocyte models using patient iPSCs were reported (Huang et al., 2011; Raval et al., 2015; Sato et al., 2016). We previously reported a skeletal muscle model of IOPD using patient-specific iPSCs (Yoshida et al., 2017). In this study, we used the same iPSC lines as used in the skeletal muscle model, and differentiated them into hepatocytes, which showed the expansion of glycogen-filled lysosomes, the hallmark of Pompe disease. We also confirmed that the glycogen accumulation was dose-dependently restored by ERT using a method of transient glucose deprivation. To our knowledge, this is the first report describing a liver model of IOPD using patient-specific iPSCs, where treatment response can be appropriately evaluated.

## Materials and Methods

### Cell Lines and Cell Culture

All human iPSC lines used in this study were described in detail in the previous report (Yoshida et al., 2017). Briefly, we generated iPSCs from fibroblasts of three healthy controls (designated as “Ctr1–3”), and three unrelated patients with IOPD (“Pom1–3”). All patients with IOPD were clinically diagnosed by the lack of GAA activity. The sequence analysis of *GAA* revealed the only single mutation of c.1880C > T in Pom1 patient, c.796C > T and c.1316T > A in Pom2, and c.1798C > T and c.2481 + 1G > A in Pom3. Then, we introduced tetracycline-inducible *MyoD* expression systems into all six iPSC lines using piggyBac vectors, and we selected two clones (“a” and “b”) with high differentiation potential into skeletal muscle from each iPSC line. All iPSC lines were cultured on mouse feeder cells in Primate ES Cell Medium (REPROCELL, Yokohama, Japan) containing 10 ng/mL of recombinant human basic fibroblast growth factor (bFGF) (Oriental Yeast, Tokyo, Japan).

### *In vitro* Hepatic Differentiation and rhGAA Rescue Experiment

For hepatic differentiation, we modified a previously reported protocol (Kajiwara et al., 2012). Briefly, iPSCs were dissociated to single cells with Accutase (Nacalai Tesque, Kyoto, Japan) and seeded on Matrigel (BD Biosciences, San Diego, CA, United States)-coated plates at the density of 1 × 10^5^ cells/cm^2^. The cells were cultured with RPMI1640 (Nacalai Tesque) containing 1 × B27 supplement (Thermo Fisher Scientific, Waltham, MA, United States), 100 ng/mL activin A (PeproTech, Rocky Hill, NJ, United States), and 50 ng/mL CHIR99021 (Merck, Darmstadt, Germany) from day 0 to day 5. Y-27632 was added for the first day, and sodium butyrate (Merck) was added at 0.5 mM from day 1 to day 4. The medium was changed daily from day 2. Next, on day 6, the culture medium was switched to knockout-DMEM (Thermo Fisher Scientific) containing 20% (vol/vol) KSR (Thermo Fisher Scientific), 1 mM L-glutamine (Thermo Fisher Scientific), 1% (vol/vol) non-essential amino acids (Thermo Fisher Scientific), 0.1 mM 2-mercaptoethanol (2-ME) (Thermo Fisher Scientific), 10 ng/mL bFGF, and 20 ng/mL Bone Morphogenetic Protein-4 (PeproTech). Finally, on day 13, the medium was replaced with hepatocyte culture medium (Lonza, Basel, Switzerland) containing 20 ng/mL hepatocyte growth factor (PeproTech) and 20 ng/mL oncostatin M (PeproTech). The medium was changed every 2 day from day 6. For the transient glucose deprivation experiment, the medium was replaced with glucose-free DMEM/Ham’s F-12 (Nacalai Tesque), 1 mM L-glutamine and 0.1 mM 2-ME for 12 h prior to the glycogen analysis. For the rhGAA rescue experiment, Myozyme (rhGAA) (Sanofi, Cambridge, MA, United States) was added to the medium for the last 3 days of differentiation.

### RNA Isolation and RT-PCR

Total RNA was isolated using Sepazol (Nacalai Tesque) according to the manufacturer’s instructions. Isolated RNA was treated with DNase and then reverse transcribed using ReverTra Ace kit (Toyobo, Osaka, Japan). Quantitative PCR for hepatic markers was performed on a StepOnePlus^TM^ instrument (Thermo Fisher Scientific) with SYBR Green dye (Thermo Fisher Scientific). PCR primers used in this study are as follows: 5′-AAATGCGTTT CTCGTTGCTT and 3′-GCCACAGGCCAATAGTTTGT for alfa-fetoprotein (AFP); 5′-CTTCCTGGGCATGTTTTTGT and 3′-TGGCATAGCATTCATGAGGA for albumin (ALB); 5′-ACA TTTACCCAAACTGTCCATT and 3′-GCTTCAGTCCCTTT CTCGTC for alfa-1 anti-trypsin (A1AT); 5′-ACCACAGTCCA TGCCATCAC and 3′-TCCACCACCCTGTTGCTGTA for glyceraldehyde-3-phosphate dehydrogenase (GAPDH).

### Periodic Acid-Schiff (PAS) Stain

Periodic acid-Schiff stain was performed with the PAS Staining Kit (Muto Pure Chemicals, Tokyo, Japan) following the manufacturer’s instructions. Briefly, cells were fixed with 10.5% (w/v) formaldehyde and treated with 1% (w/v) periodic acid for 10 min at room temperature. After the cells were washed three times with distilled water, they were treated with Schiff’s reagent for 30 min at 37°C. Staining reaction was stopped by three treatment of sulfurous acid solution. The samples were completely dried and observed with a DP73 light microscope (Olympus, Tokyo, Japan) in bright field. The cytoplasm area in each cell was manually delineated and its mean color intensity ranging 0 (white) to 255 (black) based on the 8-bit gray-scale value was calculated using Photoshop CC (version 20.0.6, Adobe Systems Inc., San Jose, CA, United States).

### Glycogen Analysis

Cultured cells were trypsinized and washed twice with PBS. Then, cell pellets were sonicated on ice in distilled water. Protein concentrations of the lysates were determined using the Pierce BCA Protein Assay Kit (Thermo Fisher Scientific). The lysates were diluted to cell type-specific protein concentrations so that the glycogen amount would be within the detection range of Glycogen Assay Kit (BioVision, Milpitas, CA, United States). Then, glycogen amounts were analyzed following the manufacturer’s instructions. Fluorescence levels were measured with EnVision Multilabel Plate Reader (PerkinElmer, Waltham, MA, United States).

### Immunofluorescence and Electron Microscopy of Cultured Cells

Hepatocytes derived from iPSCs were fixed with PBS containing with 4% (w/v) paraformaldehyde for 10 min at 4°C. Fixed samples were blocked with Blocking One (Nacalai Tesque) for 45 min at 4°C and incubated overnight at 4°C with primary antibodies diluted in 10% (v/v) Blocking One in PBS-T [PBS with 0.2% (v/v) Triton X-100 solution (Nacalai Tesque)]. The following primary antibodies were used: ALB (1:200; Bethyl Laboratories, Montgomery, TX, United States) and LAMP2 (1:100; BD Biosciences, San Diego, CA, United States). Then, the samples were washed three times with PBS-T and incubated for 1 h at room temperature with secondary antibodies diluted in 10% (v/v) Blocking One in PBS-T. The following secondary antibodies were used: Alexa Fluor 488-conjugated donkey anti-goat and anti-mouse IgG (1:500; Thermo Fisher Scientific). Nuclei were stained with DAPI (1:5000; Merck) or TO-PRO-3 (1:1000; Thermo Fisher Scientific). Samples were observed with a BZ-9000 fluorescence microscope (Keyence, Osaka, Japan) or LSM710NLO confocal microscope (Carl Zeiss, Oberkochen, Germany). For electron microscopy, samples were chemically fixed and observed by Tokai Electron Microscopy, Inc. (Nagoya, Japan).

### Statistical Analysis

Statistical analyses were performed using Scheffé’s multiple comparison method when comparing control vs. Pompe disease groups. To analyze the response to different doses of rhGAA, Williams’ multiple comparison test was used. To analyze the response of rhGAA in PAS stain, Welch’s *t*-test was used. Data was shown as mean ± standard error. ^∗^*p* < 0.05, ^∗∗^*p* < 0.01, ^∗∗∗^*p* < 0.001.

## Results

### Lysosomal Glycogen Accumulation in Hepatocytes Derived From Pom-iPSCs

**FIGURE 1:**
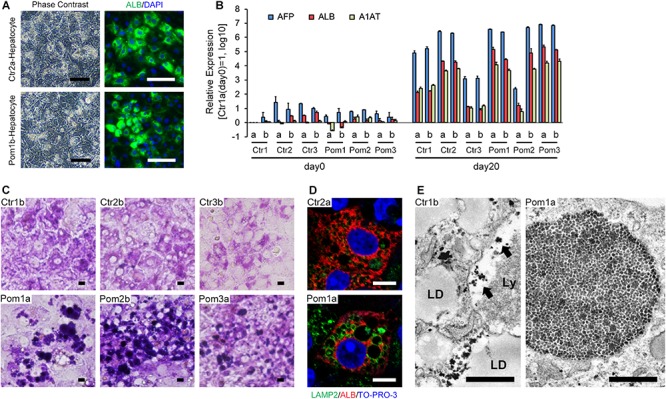
Hepatic differentiation of iPSCs and lysosomal glycogen accumulation in Pom-iPSC-derived hepatocytes. Two clones (designated as “a” and “b”) were selected from each iPSC line. **(A)** Phase contrast microscopic images (left row) and immunocytochemistry (right row) for ALB (green) in representative hepatocytes derived from Ctr- and Pom-iPSCs. Nuclei were stained with DAPI (blue). Scale bars = 100 μm. **(B)** Quantitative RT-PCR analysis for hepatic markers (AFP, blue; ALB, red; A1AT, green) at day 0 (undifferentiated iPSCs) and day 20 of hepatic differentiation in all cell lines. The graph logarithmically represents relative gene expression compared to the level of Ctr1a- iPSC at day 0 (mean ± SE, *n* = 3). GAPDH was used as an internal control. **(C)** Bright field microscopic images of PAS stain in representative hepatocytes derived from Ctr- and Pom-iPSCs. Scale bars = 10 μm. **(D)** Confocal microscopic images of immunocytochemistry for LAMP2 (green) and ALB (red) in representative hepatocytes derived from Ctr- and Pom-iPSCs. Nuclei were stained with TO-PRO-3. Scale bars = 10 μm. **(E)** Electron microscopic images in representative hepatocytes derived from Ctr- and Pom-iPSCs. Arrows indicate glycogen granules in the lysosome. Letters in the images represent the following: “LD,” lipid droplet; “Ly,” lysosome. Scale bars = 500 nm.

We differentiated iPSCs to hepatocytes according to a previously reported protocol, with a slight modification (Kajiwara et al., 2012). Differentiated cells showed the characteristics of hepatocytes, including tightly packed, polygonal and cytoplasm-rich morphology and frequent positive staining for ALB (a marker of mature hepatocytes) as well as multiple nuclei in immunofluorescence (Figure 1A). The differentiation efficiency, calculated as the ratio of ALB-positive cells to total cells, ranged from 40 to 80% (Supplementary Figure). Quantitative RT-PCR showed the up-regulation of three hepatic differentiation-related markers on differentiation day 20: AFP (a marker of hepatoblasts), ALB and A1AT (a marker of mature hepatocytes) (Figure 1B). Although we selected iPSC clones by their differentiation potential to skeletal muscle in the previous study, the majority of iPSC lines well committed to hepatic differentiation. However, there is some variety between the cell lines: Pom2a and Ctr3 were poorly differentiated to mature hepatocytes (Figure 1B). To evaluate lysosomal glycogen in hepatocytes derived from iPSCs, PAS stain (a staining method that detects polysaccharides like glycogen) and immunofluorescence for LAMP2 (a marker of lysosomes) were performed. The cytoplasm was uniformly stained in hepatocytes derived from Ctr-iPSCs; in contrast, many strongly PAS-positive round structures occupied the cytoplasm of hepatocytes derived from Pom-iPSCs (Figure 1C). These round structures ranged up to 20 μm in diameter and their surface stained for LAMP2 in immunofluorescence (Figure 1D). Electron microscopy analysis revealed numerous expanded lysosomes packed with glycogen granules in Pom-iPSC-derived hepatocytes (Figure 1E); in contrast, most glycogen granules existed freely in the cytoplasm and only a few were observed in the lysosomes in Ctr-iPSC-derived hepatocytes (arrows in Figure 1E). Electron microscopy also revealed characteristic features of hepatocytes in both groups: cell-cell connection by gap junction, abundant lipid droplets and well-developed rough-surfaced endoplasmic reticulum. These data suggested hepatocytes derived from Pom-iPSCs showed abnormal glycogen accumulation in the lysosomes.

### rhGAA Rescue for Lysosomal Glycogen Accumulation in Hepatocytes Derived From Pom-iPSCs

**FIGURE 2:**
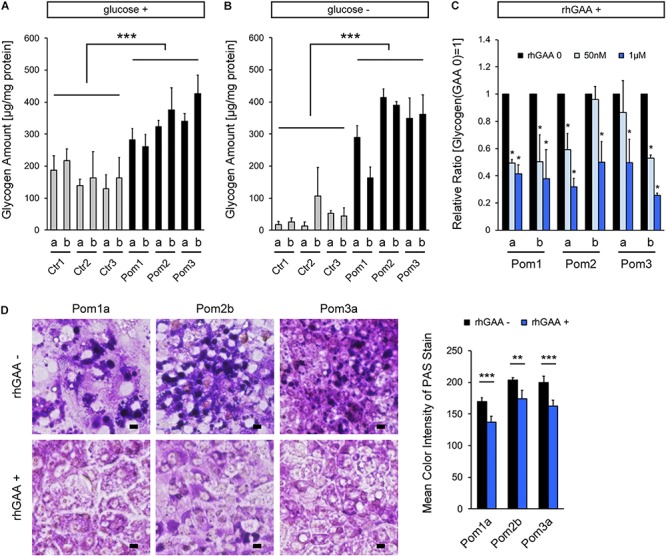
Analysis of lysosomal glycogen accumulation with transient glucose deprivation and improvement with rhGAA rescue in Pom-iPSC-derived hepatocytes. Two clones (designated as “a” and “b”) were selected from each iPSC line. **(A)** Quantitative analysis of glycogen amounts adjusted for protein amounts in normal glucose-containing culture in hepatocytes derived from iPSCs (mean ± SE, *n* = 3). **(B)** Quantitative analysis of glycogen amounts adjusted for protein amounts in hepatocytes derived from iPSCs after 12 h of culture with glucose-free media (mean ± SE, *n* = 3). **(C)** Quantitative analysis of glycogen amounts adjusted for protein amounts after 12 h of glucose deprivation with 3 days treatment of rhGAA (0, 50 nM, and 1 μM) in hepatocytes derived from Pom-iPSCs. The graph represents the relative ratio to the baseline glycogen amount (no rhGAA). Asterisks indicate a significant difference compared to the baseline (mean ± SE, *n* = 3). **(D)** Left: bright field microscopic images of PAS stain in representative hepatocytes derived from Pom-iPSCs in normal glucose-containing culture without rhGAA (upper row) and with 3 days treatment of 1 μM rhGAA (lower row). Scale bars = 10 μm. Right: mean color intensities of PAS stain in the cytoplasm of hepatocytes derived from Pom-iPSCs (*n* = 10 cells). ^∗^*p* < 0.05, ^∗∗^*p* < 0.01, ^∗∗∗^*p* < 0.001.

Quantitative analysis of glycogen amount adjusted for protein amount revealed a significantly higher level in Pom-iPSC-derived hepatocytes than Ctr (Figure 2A). Generally, cytoplasmic glycogen is broken down into glucose by two enzymes (glycogen phosphorylase and debranching enzyme); while, lysosomal glycogen is broken down only by GAA (Roach et al., 2012). In Pompe disease (lacking in GAA), only lysosomal glycogen cannot be broken down. To accurately evaluate the accumulated lysosomal glycogen, the culture medium was replaced with glucose-free medium 12 h prior to the glycogen analysis so that cytoplasmic glycogen would be consumed. After 12 h of glucose deprivation, glycogen amounts were remarkably decreased only in Ctr-iPSC-derived hepatocytes, and the difference in glycogen amounts between Ctr- and Pom-iPSC-derived hepatocytes was increased (Figure 2B). Furthermore, to evaluate the effect of rhGAA in our hepatocyte model, rhGAA was added for the last 3 days of hepatic differentiation at three different concentrations: 0, 50 nM, and 1 μM. The glycogen amounts after glucose deprivation decreased with rhGAA in a dose-dependent manner (Figure 2C). Without glucose deprivation, the total glycogen amounts were not changed with rhGAA treatment in quantitative analysis (data not shown), while abnormal expansion of glycogen-filled lysosomes observed in PAS stain almost disappeared with 3 days treatment of 1 μM rhGAA (Figure 2D). This means that accumulated lysosomal glycogen is degraded into glucose by rhGAA treatment, and the yielded glucose is used to synthesize glycogen in the cytoplasm in glucose-rich conditions.

## Discussion

In IOPD, one of the most serious clinical problems is the insufficient effect of rhGAA on skeletal muscle symptoms compared to those on the other organs including heart and liver. To investigate and solve this problem, appropriate models of multiple organs are necessary. A merit of an iPSC-based disease model is the ability to generate a multiple-lineage model from the same clone. Taking advantage of this aspect of iPSCs, we have demonstrated the recapitulation of IOPD phenotypes using patient iPSCs in two lineages, skeletal muscle in the previous study (Yoshida et al., 2017) and liver in this study. Cardiomyocyte models of Pompe disease using patient iPSCs have been also established (Huang et al., 2011; Raval et al., 2015; Sato et al., 2016). In this manner, the differences of pathomechanism among multiple lineages can be investigated by utilizing an iPSC-based disease model. However, maturity or culture conditions considerably differ between different cell types differentiated from iPSCs; thus, comparison and interpretation should be done carefully. In our models, hepatocytes differentiated from Pom-iPSCs needed more rhGAA in the rescue experiment than myocytes from Pom-iPSCs, which is opposite to the clinical situation. This might be in part attributed to the difference of the culture conditions such as the longer culture duration in hepatic differentiation. In addition, we did not evaluate a genome-wide gene expression pattern or metabolic profiles of hepatocytes derived from iPSCs, and thus we could not conclude that our model completely mimic hepatocytes in patients with IOPD. In the future, a better differentiation protocol like a three-dimension culture system may overcome this kind of limitations in iPSC-based disease modeling.

As described in the previous reports (Raval et al., 2015; Yoshida et al., 2017), a method of transient glucose deprivation enabled us to quantify the glycogen amount accumulated in the lysosomes in Pom-iPSC-derived hepatocytes, and to appropriately assess the effect of ERT. Thus, this is a useful and easy method for the appropriate evaluation of accumulated lysosomal glycogen in *in vitro* models of Pompe disease. Another intriguing aspect of our glucose deprivation experiment is that total glycogen amount was not greatly changed with or without glucose deprivation in Pom-iPSC-derived hepatocytes (Figure 2), which was also observed in our skeletal muscle model of IOPD (Yoshida et al., 2017). This means that the vast majority of glycogen was located in the lysosomes in IOPD and only a small amount of glycogen existed in the cytoplasm. This suggests two possibilities: glucose deprivation promoted glycogen transport from the cytoplasm to the lysosomes; or accumulated lysosomal glycogen negatively regulated cytoplasmic glycogen synthesis. In mice and rats, glycogen is drastically transported to the lysosomes during the starvation just after birth in the skeletal muscle and liver (Schiaffino et al., 2008), and thus iPSC-derived immature hepatocytes and myocytes may have a similar nature. However, as far as we observed with the PAS stain, the size of glycogen-filled lysosomes was not changed with or without glucose deprivation (data not shown). As for the latter possibility, an increasing number of data have recently revealed that lysosomes are not just waste disposals but also participants in some signaling pathways (Settembre et al., 2012). However, there has been no report of a pathway connecting between lysosomal glycogen and cytoplasmic glycogen metabolism. Further investigation is necessary to assess the relation of such cellular phenotypes of glycogen transport or lysosomal signaling pathways with the pathomechanism of IOPD.

In conclusion, we have successfully established an *in vitro* liver model of IOPD using patient-specific iPSCs. We have also demonstrated that our model can be used to quantitatively evaluate the response of rhGAA using a transient glucose deprivation method. This model can be a platform to elucidate the underlying disease mechanism or to screen drugs and compounds that assist rhGAA or improve the phenotype in a novel manner. Moreover, our study also suggest that an iPSC-based approach is suitable for modeling of diseases that affect multiple organs like Pompe disease.

## Data Availability Statement

The raw data supporting the conclusions of this manuscript will be made available by the authors, without undue reservation, to any qualified researcher.

## Ethics Statement

The studies involving human participants were reviewed and approved by the Kyoto University Graduate School and Faculty of Medicine, Kyoto University Hospital Ethics Committee. Written informed consent to participate in this study was provided by the participants’ legal guardian/next of kin. All experimental protocols in the study were approved by the Ethics Committee Graduate School and Faculty of Medicine Kyoto University (approval number #R0091 and #G259). The study was performed conforming to the guidelines of the Declaration of Helsinki and conducted after obtaining written informed consents.

## Author Contributions

TY designed the research and wrote the manuscript. TY and TJ performed the experiments. TY, KO, and HS analyzed the data. KO, JT, and HS supervised the research.

## Conflict of Interest

The authors declare that the research was conducted in the absence of any commercial or financial relationships that could be construed as a potential conflict of interest.
