# Supplementary material for: A Liver Model of Infantile-Onset Pompe Disease Using Patient-Specific Induced Pluripotent Stem Cells
Source: Front Cell Dev Biol. 2019 Nov 29;7:316. doi: 10.3389/fcell.2019.00316 (PMC6895003; doi:10.3389/fcell.2019.00316)
Supplement: Supplementary file 1 [file Data_Sheet_1.PDF]

Supplementary Material

The differentiation efficiency, calculated as the ratio of albumin (ALB)-positive cells to total cells, ranged from 40% to 80% in representative iPSC clones derived from both control (Ctr) and Pompe disease (Pom) groups. The number of ALB and DAPI double-positive areas and that of total DAPI-positive areas were counted in three different immunofluorescent images in each iPSC line using BZ-X Analyzer software (KEYENCE, Osaka, Japan).

Supplementary Figure

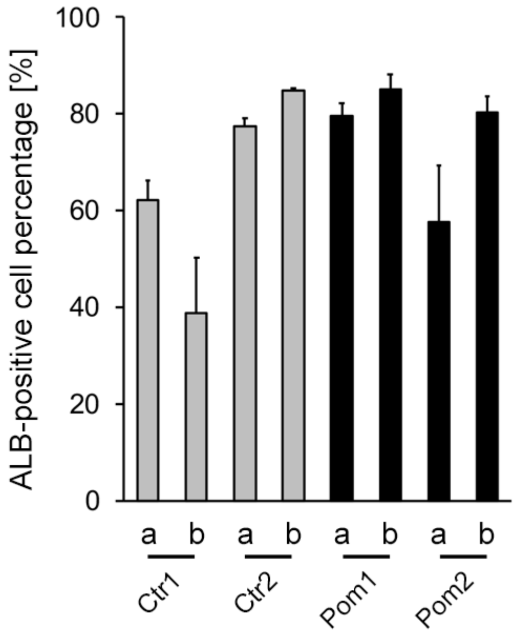

Supplementary Figure. Percentage of ALB-positive cells per total cells at day 20 of hepatic differentiation in representative iPSC lines from both Ctr and Pom groups (n=3 microscopic fields). Two clones (designated as “a” and “b”) were selected from each iPSC line.
